# Feasibility of party balloon inflation manoeuvre for haemodynamic provocation: a pilot study in healthy volunteers

**DOI:** 10.1093/ehjimp/qyaf071

**Published:** 2025-06-03

**Authors:** Kento Kito, Akihisa Kataoka, Maki Okamoto, Satoshi Nakada, Kazuyo Shirakura, Hanako Kobayashi, Ikumi Chikuda, Junichi Nishikawa, Yosei Iseki, Taiga Katayama, Hideyuki Kawashima, Takeyuki Sajima, Hirosada Yamamoto, Yusuke Watanabe, Naoyuki Yokoyama, Ken Kozuma

**Affiliations:** Division of Cardiology, Department of Internal Medicine, Teikyo University, 2-11-1 Kaga, Itabashi-ku, Tokyo 173-8605, Japan; Division of Cardiology, Department of Internal Medicine, Teikyo University, 2-11-1 Kaga, Itabashi-ku, Tokyo 173-8605, Japan; Division of Cardiology, Department of Internal Medicine, Teikyo University, 2-11-1 Kaga, Itabashi-ku, Tokyo 173-8605, Japan; Division of Cardiology, Department of Internal Medicine, Teikyo University, 2-11-1 Kaga, Itabashi-ku, Tokyo 173-8605, Japan; Department of Clinical Laboratory, Teikyo University Hospital, Tokyo, Japan; Department of Clinical Laboratory, Teikyo University Hospital, Tokyo, Japan; Department of Clinical Laboratory, Teikyo University Hospital, Tokyo, Japan; Department of Rehabilitation, Teikyo University Hospital, Tokyo, Japan; Division of Cardiology, Department of Internal Medicine, Teikyo University, 2-11-1 Kaga, Itabashi-ku, Tokyo 173-8605, Japan; Division of Cardiology, Department of Internal Medicine, Teikyo University, 2-11-1 Kaga, Itabashi-ku, Tokyo 173-8605, Japan; Division of Cardiology, Department of Internal Medicine, Teikyo University, 2-11-1 Kaga, Itabashi-ku, Tokyo 173-8605, Japan; Department of Anesthesiology, Teikyo University, Tokyo, Japan; Division of Cardiology, Department of Internal Medicine, Teikyo University, 2-11-1 Kaga, Itabashi-ku, Tokyo 173-8605, Japan; Division of Cardiology, Department of Internal Medicine, Teikyo University, 2-11-1 Kaga, Itabashi-ku, Tokyo 173-8605, Japan; Division of Cardiology, Department of Internal Medicine, Teikyo University, 2-11-1 Kaga, Itabashi-ku, Tokyo 173-8605, Japan; Division of Cardiology, Department of Internal Medicine, Teikyo University, 2-11-1 Kaga, Itabashi-ku, Tokyo 173-8605, Japan

**Keywords:** hypertrophic obstructive cardiomyopathy, patent foramen ovale, Valsalva manoeuvre, party balloon inflation manoeuvre, transthoracic echocardiography

## Abstract

**Aims:**

Left ventricular outflow tract (LVOT) obstruction is a key feature of hypertrophic obstructive cardiomyopathy (HOCM), whereas patent foramen ovale (PFO) obstruction is associated with cryptogenic stroke and other conditions. The Valsalva manoeuvre (VM) is a standard technique for diagnosing these conditions; however, its inconsistent execution can limit diagnostic accuracy. We aimed to evaluate the party balloon inflation manoeuvre (PBIM) as an alternative to VM for diagnosing HOCM and PFO by comparing their haemodynamic effects.

**Methods and results:**

In this single-centre pilot study, we conducted *in vitro* and *in vivo* experiments. The pressure characteristics of the two balloon sizes were measured in the *in vitro* experiment. In the *in vivo* study, we assessed haemodynamic changes in 25 healthy volunteers using transthoracic echocardiography. The endpoints included the left ventricular diastolic dimension (LVDd) for HOCM and the right ventricular inflow velocity-time integral (RV inflow-VTI) for PFO. PBIM significantly reduced LVDd compared with VM, indicating greater LVOT obstruction provocation (*P* < 0.01). The RV inflow-VTI was also significantly higher with PBIM, suggesting increased venous return and enhanced right-to-left shunting (*P* < 0.01). The heart rate and perceived exertion scores were higher with the PBIM, reflecting a greater physiological load.

**Conclusion:**

PBIM is a simple, effective, and reliable alternative to VM for diagnosing HOCM and PFO, offering clear visual feedback and improved diagnostic performance. Further research in patient populations is required to confirm these findings.

**Trial registration number:** UMIN000054423.

(https://center6.umin.ac.jp/cgi-open-bin/ctr/ctr.cgi?function=brows&action=brows&recptno=R000062098&type=summary&language=J).

## Graphical Abstract

**Graphical Abstract:**
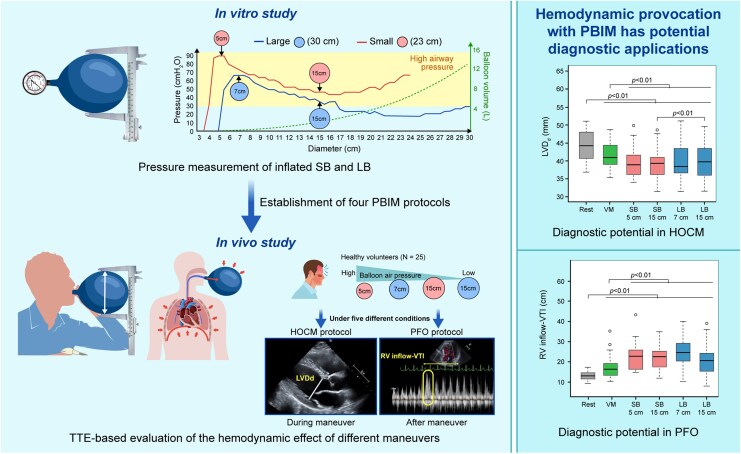

## Introduction

With the advancement of new therapeutic methods such as cardiac myosin inhibitors and patent foramen ovale (PFO) closures, the importance of provocative tests that increase venous return or pressure gradient in echocardiography is increasing in daily practice.^1,2^ Left ventricular outflow tract (LVOT) obstruction is a key feature of hypertrophic obstructive cardiomyopathy (HOCM), often causing significant symptoms and posing the risks of heart failure and death.^3,4^ In addition to established pharmacological therapies such as beta-blockers and calcium channel blockers, major therapies, such as ventricular septal myectomy, alcohol septal ablation, and dual-chamber pacing, have been used to alleviate obstruction-related symptoms. Moreover, phase 3 trials of the novel cardiac myosin inhibitor mavacamten showed promising outcomes, including reduced LVOT gradients, symptom relief, improved physical function, and decreased need for septal reduction therapy.^1^ PFO is associated with conditions such as cryptogenic stroke (paradoxical embolism), migraine, and platypnea-orthodeoxia syndrome.^5–7^ Additionally, transcatheter PFO closure effectively reduces stroke recurrence compared with medical therapy.^8–11^ These advancements highlight the growing need for an accurate and reliable diagnosis of HOCM and PFO.

The Valsalva manoeuvre (VM) is a simple, non-invasive, and standard provocative technique for assessing LVOT obstruction in HOCM and detecting right-to-left shunts through a PFO using agitated saline contrast transthoracic echocardiography (TTE).^12,13^ During VM, blood flow into the left ventricle narrows the chamber, provoking outflow tract obstruction. Conversely, increased venous return to the right atrium raises right atrial pressure, enhancing right-to-left shunting through the PFO during inhalation.^14–17^ However, uncertainty remains regarding the consistent application of these methods by both patients and examiners. A study on goal-directed VM (GDVM) in HOCM using a syringe barrel with a manometer to maintain intraoral pressure >40 mm Hg suggested that this approach may reclassify HOCM cases.^18,19^

Consequently, we developed a party balloon inflation manoeuvre (PBIM) and reported clinical cases in which PBIM was more effective than conventional VM for diagnosing HOCM and PFO.^20,21^ Unlike VM, PBIM provides a clear visual indicator of patient effort, making it easier for those who struggle with VM, such as patients with stroke, older adults, and individuals with language barriers. Hepatic vein flow assessed by TTE showed increased venous return with PBIM compared with VM.^22^ PBIM may enhance provocation visualisation, particularly when the VM is insufficient. However, haemodynamic evidence supporting PBIM's efficacy over VM for the diagnosis of PFO and HOCM is lacking. In this pilot study, we aimed to demonstrate the haemodynamic superiority of PBIM through two experiments simulating these conditions using healthy volunteers.

## Methods

### Study design

This was a single-centre pilot interventional study comprising two experiments. First, an *in vitro* experiment was conducted to understand the characteristics of party balloons by inflating two balloons of different sizes and measuring their diameters and pressures. Second, an *in vivo* experiment using TTE was performed to investigate the haemodynamic effects of PBIM in healthy adult volunteers following the protocols derived from the results of the *in vitro* experiment. All study procedures were conducted in accordance with the Declaration of Helsinki and approved by the Teikyo University Medical Research Ethics Committee (reference number TEIDAI 23-133, approved on 23 December 2023). All the participants provided written informed consent.

### In vitro experiment

Two balloons of different sizes were inflated in 5 mm diameter increments using a vernier calliper, and the corresponding pressures were measured. Two commercially available balloons of different sizes (Daiso Industries Co., Ltd., Higashihiroshima, Japan) were 23 and 30 cm in maximum diameter, referred to as the smaller balloon (SB) and larger balloon (LB), respectively. The LBs were the same as those used in previous case reports.^20^

### In vivo experiment

**Figure 1:**
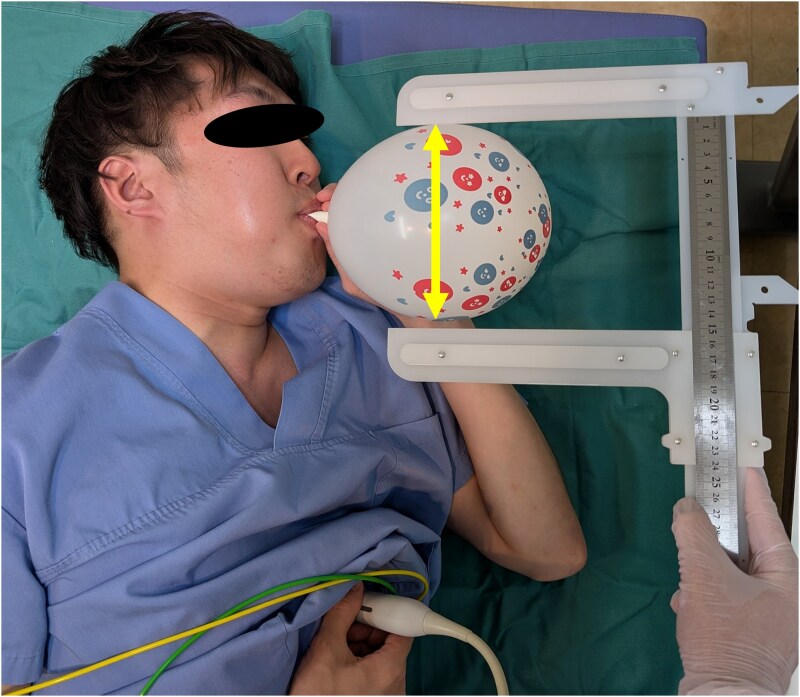
Participants performing PBIM. TTE was performed in the left lateral decubitus position while the participant inflated the party balloon according to the established protocols. The arrow indicates the diameter of the balloon measured using the vernier calliper. TTE, transthoracic echocardiography.

We studied healthy adult health worker volunteers who were physicians, cardiology fellows, residents, attending doctors, and sonographers at our hospital (Teikyo University Hospital, Tokyo, Japan) between January and August 2024. Clinical data, including age, sex, and body surface area, were collected. All participants underwent standard two-dimensional B-mode and Doppler TTE in the left lateral decubitus position and conventional parameters such as left ventricular diastolic-dimension (LVDd), left ventricular ejection fraction, left atrial volume, E/A, E/e′, grade of mitral regurgitation, and tricuspid regurgitation were measured according to the guidelines of American Society of Echocardiography/European Association of Cardiovascular Imaging.^23,24^ The images and acquired parameters were obtained by experienced physicians using a Philips ultrasound system (EPIQ CVx Cardiac Ultrasound with X5-1 xMATRIX array transducer, Philips, Andover, Massachusetts, United States) (*Figure 1*).

### Endpoints in HOCM and PFO protocol

**Figure 2:**
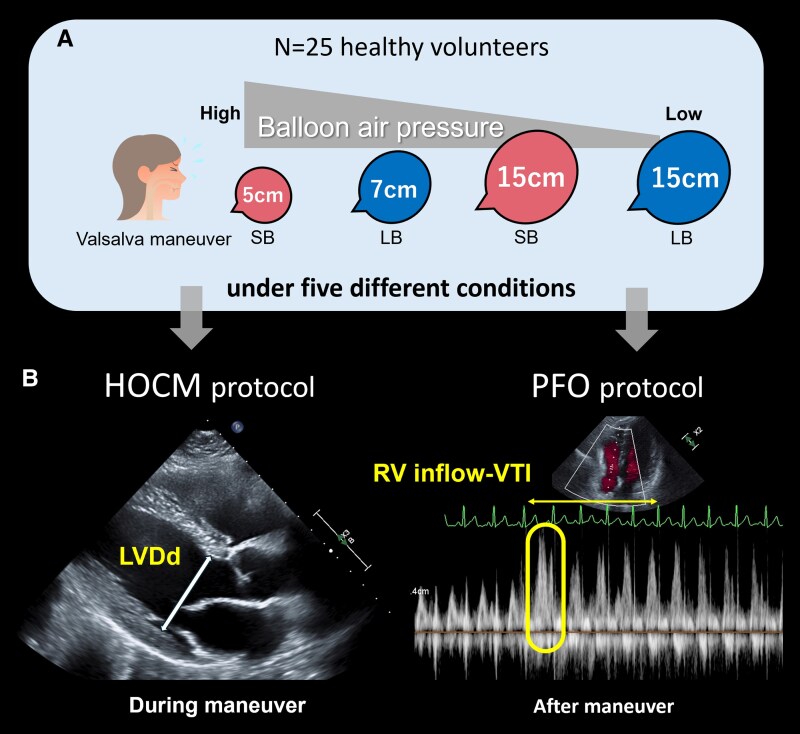
In vivo experiment design. We established four PBIM protocols: we inflated the SB to a diameter of 5 cm and the LB to 7 cm to achieve maximum pressure for each balloon. Then, both balloons were made to a diameter of 15 cm. We assessed the left ventricular end-diastolic dimension (LVDd) during the manoeuvre and the right ventricular inflow velocity-time integral (RV inflow-VTI) after the manoeuvre using TTE. LB, larger balloon; LVDd, left ventricular end-diastolic dimension, PBIM; party balloon inflation manoeuvre; RV, right ventricle; SB, smaller balloon; VTI, velocity-time integral.

In the *in vivo* experiment, LVDd, representing the haemodynamic changes in the HOCM, was assessed during VM and PBIM at the designated diameters determined from the experiment. A reduction in preload caused by VM or PBIM, as reflected by a decrease in LVDd, is associated with LVOT obstruction.^25^ For PBIM, balloon inflation was maintained for at least 5 s using a designated balloon size. This procedure is referred to as the HOCM protocol (*Figure 2B*, left panel).

Meanwhile, the maximal right ventricular (RV) inflow-velocity time integral (VTI), representing a venous return to the right atrium, was assessed over five cardiac cycles immediately following the release of VM or PBIM. These parameters were recorded during the resting phase, which we referred to as the PFO protocol (*Figure 2B*, right panel).

### Other clinical parameters

In the HOCM protocol, heart rates were collected during the VM, PBIM, and rest. Exertion experienced during the manoeuvre was recorded using the Borg rating of the perceived exertion scale, from zero (very light activity) to ten (maximum effort).

### PBIM vs. GDVM

To compare GDVM, as recommended for echocardiographic evaluation of LVOT obstruction,^18,19^ with the PBIM HOCM protocol, an additional study was conducted. Twelve healthy adult healthcare worker volunteers were enrolled. For the GDVM protocol, participants performed the manoeuvre by blowing into a syringe barrel connected to a manometer via rubber tubing (see Supplementary data online, *Figure S1*) and maintaining an intraoral pressure exceeding 40 mm Hg, which was equivalent to about 54 cm of H_2_O, for more than 10 s, following the established protocol.^18,19^

The primary endpoint for this comparison was the left ventricular end-diastolic volume (LVEDV) during the manoeuvre, reflecting preload reduction relevant to HOCM assessment. To minimize measurement bias, both the PBIM HOCM protocol and the GDVM protocol were performed using three-dimensional (3D)-TTE. The acquired 3D-TTE image datasets were analysed using dynamic heart model software (Philips, Andover, Massachusetts, United States), which generated left ventricular volume–time curves and allowed for accurate calculation of LVEDV.

### Statistical analyses

We described the participants’ characteristics using medians and interquartile ranges for continuous variables and numbers and percentages for categorical variables. Differences between the results of the different protocols were analysed using Friedman's test for RV inflow-VTI, LVDd, heart rate, and Borg RPE scale. Subsequently, the paired *t*-test, Wilcoxon signed-rank test, and the Bonferroni–Holm method were used to determine whether there were significant differences in the diagnostic performance of the protocols. For reproducibility (interobserver variability) of the endpoint, LVDd and RV inflow-VTI were assessed using Pearson's correlation coefficient and Bland–Altman analysis.^26,27^ Statistical significance was set at *P* < 0.05. All analyses were performed using R software (version 4.3.2; The R Foundation for Statistical Computing).

## Results

### In vitro experiment

**Figure 3:**
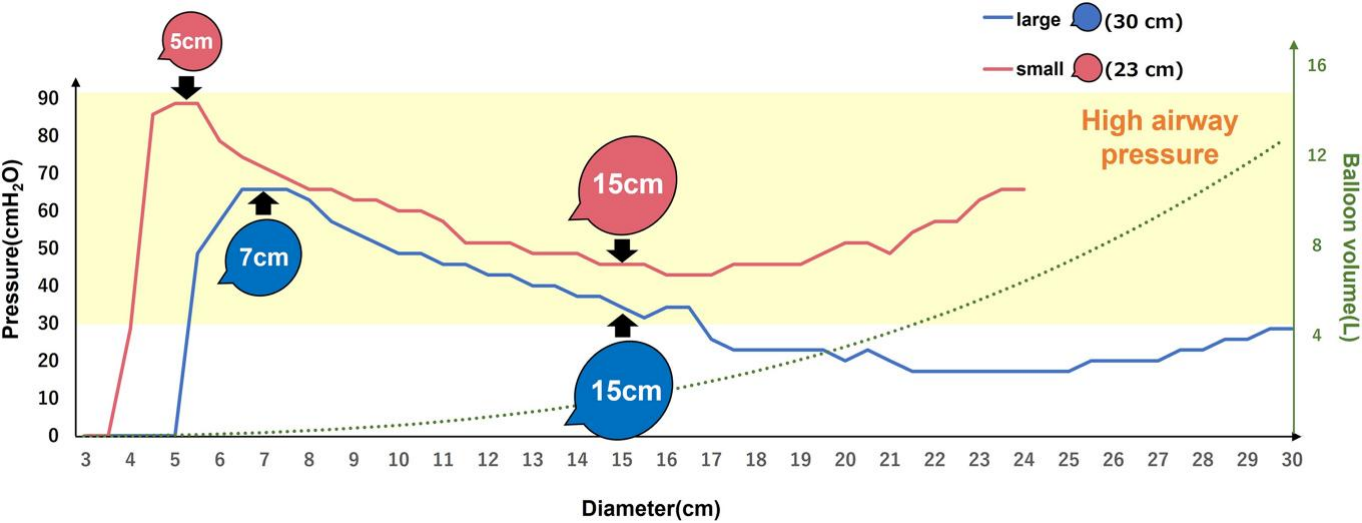
In vitro experiment: the characteristics of the balloons. The pink line represents the SB, while the blue line represents the LB. The airway pressure is consistently higher for the SB than for the LB, with the highest pressure observed when the SB reaches a diameter of 5 cm and the LB reaches 7 cm. At a diameter of 15 cm, the airway pressure stabilizes, with the LB showing a pressure of 35 cm of H₂O. This indicates a significant increase in airway pressure. LB, larger balloon; SB, smaller balloon.

We generated a graph depicting the characteristics of the balloons (*Figure 3*) from the *in vivo* experiments. The pink and blue dashed lines represent SB and LB, respectively. Air pressure was consistently higher in the SB than in the LB. The highest pressure was observed when the SB reached a diameter of 5 cm (62 cm of H_2_O) and the LB reached 7 cm (46 cm of H_2_O). During inflation, multiple breaths were required for both balloons to achieve a volume of ∼2 L and a diameter of 15 cm. At this point, the air pressure stabilized, with the LB exhibiting an air pressure of 35 cm of H_2_O.

### In vivo experiment

Based on these results, four PBIM protocols were established and tested in an *in vivo* experiment: inflating the SB to a diameter of 5 cm and the LB to a diameter of 7 cm as the maximum pressure for each balloon and inflating both balloons to a diameter of 15 cm as the stabilized pressure (*Figures 1 and 3*). Overall, 25 participants {median age, 32 [interquartile range (IQR): 29–37] years; 80% male} completed the protocol (*Table 1*). The baseline echocardiographic parameters, which demonstrated no abnormalities and normal cardiac function in all participants, are shown in *Table 1*.

**Table 1:** Participant characteristics and baseline echocardiographic parameters

Age, years	32.0 (29–37)
Male, %	20 (80)
Weight, kg	67.0 (60–76)
Height, cm	173.0 (168–177)
BSA, cm^2^	1.79 (1.69–1.91)
Rest HR, bpm	67.0 (61.5–74)
LVDd, mm	44.3 (40.7–47.9)
LVDs, mm	29.3 (26.2–31.9)
LVEDV index, mL/m^2^	43.2 (38.9–49.9)
LVESV index, mL/m^2^	17.5 (14.5–23.3)
LVEF, %	61.5 (55.1–63.9)
LAV index, mL/m^2^	17.4 (13.2–21.4)
E/e′	4.4 (3.9–5.3)
MR: none/trace/>mild	20 (80)/5 (20)/0 (0)
TR: none/trace/>mild	22 (88)/3 (12)/0 (0)

Values are median (IQR) or *n* (%).

BP, blood pressure; BSA, body surface area; E, transmitral peak E-Wave velocity; e′, peak diastolic annular velocity; HR, heart rate; LAV, left atrial volume; LVDd, left ventricular end-diastolic dimension; LVDs, left ventricular diastolic diameter; LVEF, left ventricular ejection fraction; MR, mitral regurgitation; TR, tricuspid regurgitation.

### HOCM protocol

**Figure 4:**
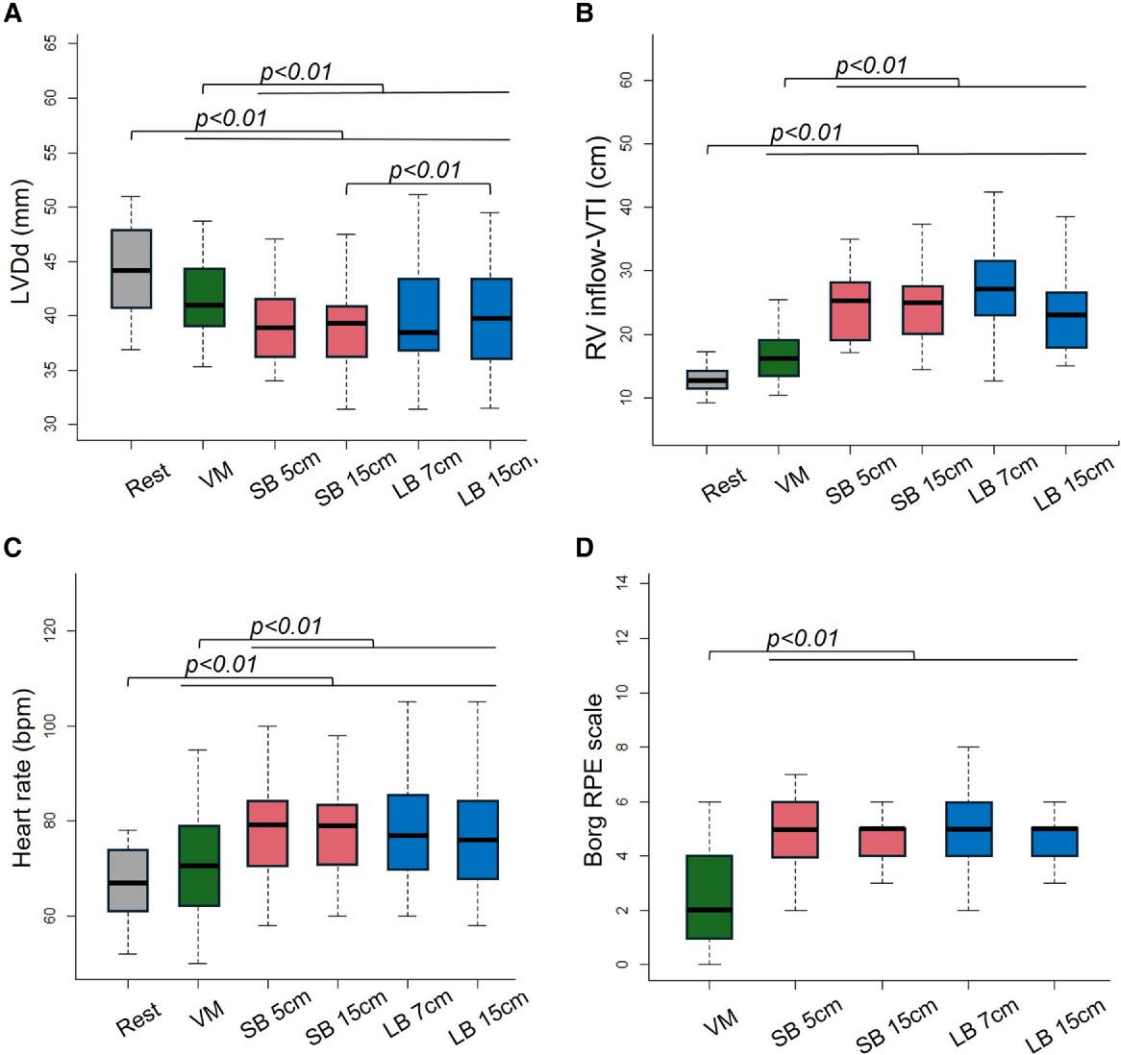
Comparison of measurements across conditions. Within each box plot, the horizontal black lines denote the median values. The boxes extend from the 25th to the 75th percentile of each group's distribution of values. The vertical lines represent adjacent values (i.e. the most extreme values within 1.5 times the IQR of the 25th and 75th percentiles of each group). Dots denote observations outside the range of adjacent values (outliers). (*A*) Comparison of LVDd at rest, during the VM, and across all PBIM protocols. *P*-values of multiple paired *t*-tests are displayed. (*B*) Comparison of RV inflow-VTI during rest, VM, and all PBIM protocols. *P*-values of pairwise comparisons using paired Wilcoxon tests are displayed. (*C*) Comparison of heart rate during rest, VM, and PBIM protocols. P-values of multiple paired t-tests are displayed. (*D*) Comparison of Borg rating of perceived exertion (RPE) scale during VM and PBIM protocols. *P*-values of pairwise comparisons using paired Wilcoxon tests are displayed. LB, larger balloon, LVDd, left ventricular end-diastolic diameter; PBIM, party balloon inflation manoeuvre; RPE, rating of perceived exertion; RV, right ventricular; SB, smaller balloon; VM, Valsalva manoeuvre; VTI, velocity-time integral.

Both VM and PBIM resulted in a reduction in LVDd compared with that in the resting state (*P* < 0.01). Notably, the LVDd was significantly smaller under all PBIM protocols [median 36.4 (IQR: 34.0–38.8) for SB 5 cm, median 36.3 (IQR: 31.4–39.3) mm for SB 15 cm, median 37.0 (IQR: 31.4–38.6) mm for LB 7 cm, and median 36.2 (IQR: 31.5–39.8) mm for LB 15 cm, respectively] compared with the VM [median 39.2 (IQR: 35.3–41.0) mm, all *P* < 0.01] (*Figure 4A*).

### PFO protocol

Both the VM and PBIM groups showed higher RV inflow VTI than they did in the resting state (both *P* < 0.01). The RV inflow VTI was ∼1.5 times larger and significantly higher under all PBIM protocols [median 19.4 (IQR: 17.1–25.5) for SB 5 cm, median 20.3 (IQR: 14.4–25.2) mm for SB 15 cm, median 23.3 (IQR: 12.6–27.3) mm for LB 7 cm, and median 18.0 (IQR: 15.1–23.3) mm for LB 15 cm, respectively] compared with that in the VM [median 13.6 (IQR: 10.5–16.2)] (all *P* < 0.01). When comparing the results of all PBIM protocols, no significant differences were observed (*Figure 4B* and *Table 2*).

**Table 2:** Comparison of LVDd, RV inflow-VTI, heart rate, and borg RPE scale during rest across conditions

	Rest	VM	SB 5cm	SB 15cm	LB 7cm	LB 15cm	*P*-value
LVDd (mm)	44.3 (40.7–47.9)	41.0 (39.2–44.4)*	38.8 (36.4–41.4)*†	39.3 (36.3–40.8)*†	38.6 (37.0–43.4)*†	39.8 (36.2–43.3)*†‡	<0.01
RV inflow-VTI (cm)	12.3 (11.8–14.3)	16.2 (13.6–19.1)*	25.5 (19.4–28.2)*†	25.2 (20.3–27.5)*†	27.3 (23.3–31.6)*†	23.3 (18.0–26.4)*†	<0.01
Heart rate	67 (61.5–74.0)	71 (62.5–79.0)*	79 (70.5–84.0)*†	79 (71.0–83.5)*†	77 (70.0–85.5)*†	76 (68.0–84.0)*†‡	<0.01
Borg RPE scale	N/A	2 (1–4)	5 (4–6)†	5 (4–5)†	5 (4–6)†	5 (4–5)†	<0.01

Values are median (IQR).

*P* value denotes significant difference across groups. *P < 0.01 vs. Rest. †*P* < 0.01 vs. VM. ‡*P* < 0.01 vs. SB 15 cm.

HR, heart rate; LB, larger balloon; LVDd, left ventricular end-diastolic dimension; N/A, not applicable, RPE, Rating of Perceived Exertion, RV inflow-VTI, right ventricular inflow-velocity time integral; SB, smaller balloon; VM, valsalva manoeuvre.

### Other clinical parameters

The heart rate did not increase significantly with the VM [median 71 (IQR: 62.5–79.0)] compared with the rest condition [median 67.0 (IQR: 61.5–74.0)], whereas it rose significantly with the PBIM [median 79 (IQR: 70.5–84.0) for SB 5 cm, median 79 (IQR: 71.0–83.5) mm for SB 15 cm, median 77 (IQR: 70.0–85.5) mm for LB 7 cm, and median 76 (IQR: 68.0–84.0) mm for LB 15 cm, respectively] (all *P* < 0.01). Moreover, compared with VM, PBIM resulted in a significantly greater increase (*P* < 0.01) (*Figure 4C* and *Table 2*). In all PBIM protocols, the Borg rating of the perceived exertion scale scores was higher than that observed during VM (*P* < 0.01) (*Figure 4D* and *Table 2*).

### Interobserver variability

The inter-observer variability of endpoint measurements, such as LVDd and RV inflow-VTI, is summarized in *Table 3*. The correlation coefficient of inter-observer variation between the two analysers showed good positive correlations for all measurements (*P* < 0.01).

**Table 3:** Inter-observer variability of endpoint measurements

LVDd (mm)		RV inflow-VTI (cm)	
Correlation coefficient	Mean differences ± 1.96SD	Correlation coefficient	Mean differences ± 1.96SD
0.88, *P* < 0.01	0.61 ± 2.25	0.92, *P* < 0.01	0.33 ± 3.61

LVDd, left ventricular end-diastolic dimension; RV, right ventricle; SD, standard deviation; VTI, velocity-time integral.

### PBIM vs. goal-directed VM

The participant characteristics and baseline echocardiographic parameters are shown in Supplementary data online, *Table S1*. Based on the results of the *in vitro* experiment, the PBIM HOCM protocol adopted the use of the SB 5 cm and the LB 7 cm, as these inflation sizes produced the highest airway pressures. Both PBIM protocols and GDVM resulted in a reduction in LVEDV [median 95.5 (IQR: 85.5–100.8) for SB 5 cm, median 96.5 (IQR: 84.0–109.5) mm for LB 7 cm, and median 97.5 (IQR: 91.0–106.3) mL for GDVM, respectively] compared with that in the resting state [median 128.5 (IQR: 120.0–136.8) mL] (*P* < 0.01 for SB 5 cm and *P* < 0.05 for LB 7 cm and GDVM). There were no significant differences among PBIM protocols and GDVM (see Supplementary data online, *Figure S2* and *Table 4*). In addition, there were no significant differences in Borg RPE scale between PBIM protocols and GDVM.

**Table 4:** Comparison of LVEDV and borg RPE scale during rest, PBIM, and GDVM

	Rest	SB 5cm	SB 7cm	GDVM	*P*-value
LVEDV (mL)	128.5 (120–136.8)	95.5 (85.5–100.8)*	96.5 (84.0–109.5)†	97.5 (91.0–106.3)†	<0.01
Borg RPE scale	N/A	5 (4–6)	5 (4–6)	5 (4.75–6.25)	0.73

Values are median (IQR).

*P* value denotes significant difference across groups. *P < 0.01 vs. Rest. †*P* < 0.05 vs. Rest.

LB, larger balloon; LVEDV, left ventricular end-diastolic volume; N/A, not applicable, RPE, Rating of Perceived Exertion, SB, smaller balloon; VM, valsalva manoeuvre.

## Discussion

This study demonstrated that PBIM appears to be a feasible and promising alternative to the conventional VM or GDVM for haemodynamic provocation in healthy volunteers. Therefore, PBIM may be an effective and reliable alternative to the conventional VM for diagnosing HOCM and PFO; however, further validation in patient populations is needed. PBIM elicited significantly greater haemodynamic responses, including reduced LVDd and increased RV inflow-VTI, compared with VM. These findings suggest that PBIM provides superior provocation for LVOT obstruction and enhanced venous return, facilitating the detection of right-to-left shunting in PFO. Additionally, PBIM offered clear visual feedback, making it easier to standardize and perform, particularly for individuals who face challenges with VM. This novel approach addresses the limitations of VM while maintaining simplicity and accessibility, highlighting its potential to improve diagnostic accuracy in clinical settings. Notably, the participants in this study were physicians or sonographers proficient in performing VM. Therefore, the effectiveness of PBIM compared with VM may be more apparent in patients who often have difficulty performing VM. However, further studies are needed to confirm its feasibility and superiority in older or less healthy individuals.

The mechanism underlying the more pronounced physiological effects of PBIM compared with those of the VM is as follows. PBIM can sustain higher intrathoracic pressure for a longer duration than VM, thereby more effectively impeding venous return and resulting in a greater reduction in preload.^21^ During inspiration to further inflate the party balloon, intrathoracic pressure decreases transiently to a negative value, facilitating increased venous return to the right atrium. Consequently, in patients with a PFO, such augmented venous return generates a right-to-left atrial pressure gradient, potentially promoting intracardiac shunting.^20,22^

### Obstructive hypertrophic cardiomyopathy

Drag forces in the hyperdynamic left ventricle led to systolic anterior motion of the mitral valve leaflet, and the combination of anatomical narrowing of the LVOT and abnormal blood flow vectors results in LVOT obstruction.^25^ Obstruction is dynamic and depends on various variables, including preload, afterload, and contractility. The VM is extensively used as a mechanism for inducing LVOT obstruction because of its ease of use. During the strain phase of the provocative manoeuvre, reduced venous return results in a decreased left ventricular filling diminished left ventricular end-systolic volume, and an increased likelihood of systolic anterior motion of the mitral valve and septal contact, with consequent LVOT obstruction.

In our study, LVDd during manoeuvre was smaller in all PBIM protocols than at rest and during VM, suggesting that PBIM reduces blood flow to the left ventricle more than VM and decreases left ventricular end-systolic volume, thereby narrowing the LVOT and leading to a more effective diagnosis of LVOT obstruction in patients with HOCM. Therefore, PBIM may induce a higher peak of LVOT pressure and reclassify more patients from non-obstructive to HOCM than conventional VM.

Exercise stress testing is also widely used for the comprehensive evaluation of patients.^28^ Increased contractility and exercise can induce LVOT obstruction, which is a key element of this diagnostic method. However, some patients who are candidates for intervention may be unable to undergo exercise echocardiography due to frailty or comorbidities. In such cases, PBIM may be an alternative provocation method when VM fails to induce a peak LVOT pressure of 50 mmHg. However, the differences between PBIM and exercise stress should be evaluated in future clinical investigations involving patients with HOCM.

The use of a syringe barrel connected to an aneroid manometer as GDVM, a method recommended by current professional societies for the reclassification of HOCM, may be a viable option.^18,19^ Specifically, *in vivo* experiments demonstrated that the air pressure at SB 5 cm and LB 7 cm exceeded the target orbital inner pressure of the GDVM. Although the PBIM inflation time in this protocol is 5 s—half the duration of positive airway pressure compared with the 10 s required for GDVM—PBIMs resulted in a comparable reduction in LVEDV. This finding suggests that PBIM may have at least a similar capacity to provoke LVOT obstruction. Moreover, GDVM is complex due to the need for device assembly, whereas PBIM offers greater simplicity and cost-effectiveness, requiring only a single ordinary balloon.

### Patent foramen ovale

Agitated saline contrast TTE is the gold standard for detecting right-to-left shunting,^29^ and an adequately performed VM that increases venous return and right atrium pressure is crucial for diagnosis. False negatives have been reported in PFO detection cases where VM is not properly executed, highlighting the need for more reliable provocation methods for accurate PFO diagnosis.^17^ In our study, after the manoeuvre, RV inflow-VTI was higher in all PBIM protocols compared with rest and VM, indicating an increased venous return to the right atrium, which elevated the right atrial pressure more than VM did. This may result in enhanced right-to-left shunting through the PFO and improve its diagnosis. To validate this PBIM advantage in real-world practice, we are currently conducting a multi-centre registry study: the impact of PBIM during saline contrast transthoracic echocardiography for detecting patent foramen ovale (INFLATE-PFO) registry (UMIN clinical trials registry: R000058907).

### Other superiorities compared with the conventional VM

An adequate VM is crucial for diagnosing HOCM and PFO.^13,30^ However, the VM has the problem that data depends on the skill and effort of the patient and the examiner, and there is no standardized method. In contrast, PBIM is straightforward for clinicians to explain to patients and enables an objective assessment of the manoeuvre through direct observation of balloon inflation. In vitro studies have demonstrated that an inflated balloon consistently applies airway pressure ranging from 30 to 80 cm of H_2_O, which is considered high airway pressure to access LVOT obstruction in patients with HOCM^31^ Additional findings support the strength of PBIM: the reflexive increase in heart rate in response to decreased aortic pressure during the manoeuvre is well-documented. The elevated heart rate observed during PBIM indicates that it provides a stronger and more reliable physiological load than the VM. Furthermore, higher scores on the Borg rating of the perceived exertion scale confirmed that PBIM imposed a greater load than did VM. Consequently, PBIM has already been implemented and utilized in many institutions across Japan, underscoring its practical advantages.

### Recommended size of the balloon and inflation

The size or type of balloon may not have significantly affected the diagnosis in this study. However, SBs, or balloons inflated to smaller sizes, may be more advantageous. This is because of the observed characteristics of the balloons, which showed a consistently higher air pressure for the SB than they did for the LB, with peak pressure occurring early in the inflation process. This suggests that examinees do not need to inflate the balloons to a large size to achieve a reliable diagnosis, which is particularly beneficial for individuals who struggle to perform VM correctly because of frailty.

### Study limitations

The present study was an exploratory single-centre pilot study involving physically active, healthy volunteers to examine physiological response rather than diagnosing existing conditions. In addition, the sample size was small with a lack of actual patients with confirmed HOCM or PFO, which limits direct clinical applicability of the findings. Further research is required to assess the generalisability of the PBIM in diverse patient populations, particularly among those with cardiovascular or respiratory conditions, such as patients with HOCM or PFO, and to confirm its clinical utility. Furthermore, this study was conducted exclusively in highly educated healthcare professionals, introducing a potential selection bias. The applicability and reproducibility of PBIM in the general population, particularly among individuals unfamiliar with respiratory manoeuvres, remain to be validated in future studies. A multi-centre clinical trial is currently underway (INFLATE-PFO registry), which may help address the issue.

Third, in the PFO protocol, PBIM was compared only with the conventional VM but not with the alternative provocation method, such as VM plus abdominal compression—which is considered a standard provocative method for detecting right-to-left shunts in patients with PFO^32^— due to concerns about invasiveness for participants. Exercise stress testing, which is used in HOCM patients to enhance haemodynamic changes,^28^ was not performed for the same reason described in the discussion. Fourth, although we measured airway pressure during balloon inflation to determine the pressure characteristics for each balloon size and to establish appropriate target diameters, we did not directly assess whether the intrathoracic pressure achieved was consistent across individuals. Therefore, the standardization of intrathoracic pressure during PBIM remains uncertain and should be further investigated. Finally, PBIM requires more effort than VM, which might be challenging for older patients or those with frailty. Therefore, alternative approaches may be needed for older patients or those with frailty.

## Conclusions

Our findings suggest that PBIM induces greater haemodynamic changes than VM, regardless of balloon type or size, and may enhance diagnostic accuracy in HOCM and PFO. Its simplicity and clear visual feedback make the PBIM particularly useful for patients who struggle with VM. These findings suggest that PBIM is a reliable and practical alternative to VM and warrants further validation in diverse patient populations.

## Supplementary Material

qyaf071_Supplementary_Data

## Data Availability

The data, analytic methods, and study materials will be made available to other researchers for the purposes of reproducing these results or replicating these procedures from the corresponding author upon reasonable request.

## Lead author biography

Dr Kento Kito graduated from Teikyo University, Japan. He currently works as a clinical staff in the Department of Cardiology, Teikyo University Hospital, Tokyo, Japan. He specializes in transthoracic and transoesophageal echocardiography, especially in structural heart disease.

## References

[1] Olivotto I, Oreziak A, Barriales-Villa R, Abraham TP, Masri A, Garcia-Pavia P et al Mavacamten for treatment of symptomatic obstructive hypertrophic cardiomyopathy (explorer-HCM): a randomised, double-blind, placebo-controlled, phase 3 trial. Lancet 2020;396:759–69. 32871100 10.1016/S0140-6736(20)31792-X

[2] Mojadidi MK, Zaman MO, Elgendy IY, Mahmoud AN, Patel NK, Agarwal N et al Cryptogenic stroke and patent foramen ovale. J Am Coll Cardiol 2018;71:1035–43. 29495983 10.1016/j.jacc.2017.12.059

[3] Semsarian C, Ingles J, Maron MS, Maron BJ. New perspectives on the prevalence of hypertrophic cardiomyopathy. J Am Coll Cardiol 2015;65:1249–54. 25814232 10.1016/j.jacc.2015.01.019

[4] Maron MS, Finley JJ, Bos JM, Hauser TH, Manning WJ, Haas TS et al Prevalence, clinical significance, and natural history of left ventricular apical aneurysms in hypertrophic cardiomyopathy. Circulation 2008;118:1541–9. 18809796 10.1161/CIRCULATIONAHA.108.781401

[5] Lip GYH, Lane DA, Lenarczyk R, Boriani G, Doehner W, Benjamin LA et al Integrated care for optimizing the management of stroke and associated heart disease: a position paper of the European Society of Cardiology council on stroke. Eur Heart J 2022;43:2442–60. 35552401 10.1093/eurheartj/ehac245 PMC9259378

[6] Kerut EK, Norfleet WT, Plotnick GD, Giles TD. Patent foramen ovale: a review of associated conditions and the impact of physiological size. J Am Coll Cardiol 2001;38:613–23. 11527606 10.1016/s0735-1097(01)01427-9

[7] Khessali H, Mojadidi MK, Gevorgyan R, Levinson R, Tobis J. The effect of patent foramen ovale closure on visual aura without headache or typical aura with migraine headache. JACC Cardiovasc Interv 2012;5:682–7. 22721665 10.1016/j.jcin.2012.03.013

[8] Carroll JD, Saver JL, Thaler DE, Smalling RW, Berry S, MacDonald LA et al Closure of patent foramen ovale versus medical therapy after cryptogenic stroke. N Engl J Med 2013;368:1092–100. 23514286 10.1056/NEJMoa1301440

[9] Saver JL, Carroll JD, Thaler DE, Smalling RW, MacDonald LA, Marks DS et al Long-term outcomes of patent foramen ovale closure or medical therapy after stroke. N Engl J Med 2017;377:1022–32. 28902590 10.1056/NEJMoa1610057

[10] Mas J-L, Derumeaux G, Guillon B, Massardier E, Hosseini H, Mechtouff L et al Patent foramen ovale closure or anticoagulation vs. antiplatelets after stroke. N Engl J Med 2017;377:1011–21. 28902593 10.1056/NEJMoa1705915

[11] Søndergaard L, Kasner SE, Rhodes JF, Andersen G, Iversen HK, Nielsen-Kudsk JE et al Patent foramen ovale closure or antiplatelet therapy for cryptogenic stroke. N Engl J Med 2017;377:1033–42. 28902580 10.1056/NEJMoa1707404

[12] Bernard S, Churchill TW, Namasivayam M, Bertrand PB. Agitated saline contrast echocardiography in the identification of intra- and extracardiac shunts: connecting the dots. J Am Soc Echocardiogr 2020;34:1–12. 10.1016/j.echo.2020.09.013 34756394

[13] Ommen SR, Mital S, Burke MA, Day SM, Deswal A, Elliott P et al 2020 AHA/ACC guideline for the diagnosis and treatment of patients with hypertrophic cardiomyopathy: a report of the American college of cardiology/American heart association joint committee on clinical practice guidelines: a report of the American College of Cardiology/American Heart Association joint committee on clinical practice guidelines. Circulation 2020;142:e558–631. 33215931 10.1161/CIR.0000000000000937

[14] Hamilton WF . Physiologic relationships between intrathoracic, intraspinal, and arterial pressures. J Am Med Assoc 1936;107:853–6.

[15] Zhang X-Y, Cao T-S, Yuan L-J. The mechanics of left ventricular filling during the strain phase of the Valsalva maneuver in healthy subjects. Am J Med Sci 2013;346:187–9. 23114199 10.1097/MAJ.0b013e31826af7de

[16] Braunwald E, Oldham HN Jr, Ross J Jr, Linhart JW, Mason DT, Fort L 3rd. The circulatory response of patients with idiopathic hypertrophic subaortic stenosis to nitroglycerin and to the Valsalva maneuver. Circulation 1964;29:422–31. 14128830 10.1161/01.cir.29.3.422

[17] Cheng TO . The proper conduct of Valsalva maneuver in the detection of patent foramen ovale. J Am Coll Cardiol 2005;45:1145–6. 15808778 10.1016/j.jacc.2004.12.054

[18] Kumar S, Van Ness G, Bender A, Yadava M, Minnier J, Ravi S et al Standardized goal-directed Valsalva maneuver for assessment of inducible left ventricular outflow tract obstruction in hypertrophic cardiomyopathy. J Am Soc Echocardiogr 2018;31:791–8. 29573929 10.1016/j.echo.2018.01.022

[19] Bavishi A, Soutar M, Kurnides M, Speer H, Oyarce G, Bryde R et al Goal-directed versus self-directed Valsalva maneuver in patients with hypertrophic cardiomyopathy on cardiac myosin inhibitor therapy. JACC Adv 2025;4:101531. 39886312 10.1016/j.jacadv.2024.101531 PMC11780103

[20] Kataoka A, Kito K, Sajima T, Watanabe Y, Kozuma K. Party balloon inflation maneuver during saline contrast transthoracic echocardiography to detect patent foramen ovale. JACC Case Rep 2022;4:102–4. 35106494 10.1016/j.jaccas.2021.10.012 PMC8784720

[21] Kito K, Kataoka A, Katayama T, Watanabe Y, Kozuma K. Left ventricular outflow tract obstruction induced by party balloon inflation manoeuvre in transthoracic echocardiography. Eur Heart J Case Rep 2023;7:ytad156. 37090750 10.1093/ehjcr/ytad156 PMC10118625

[22] Kataoka A, Kito K, Shirakura K, Katayama T, Kozuma K. Increasing venous return blood flow to the right atrium using the party balloon inflation maneuver. JACC Case Rep 2023;22:101997. 37790771 10.1016/j.jaccas.2023.101997 PMC10544291

[23] Lang RM, Badano LP, Mor-Avi V, Afilalo J, Armstrong A, Ernande L et al Recommendations for cardiac chamber quantification by echocardiography in adults: an update from the American Society of echocardiography and the European Association of cardiovascular imaging. J Am Soc Echocardiogr 2015;28:1–39.e14. 25559473 10.1016/j.echo.2014.10.003

[24] Lang RM, Badano LP, Mor-Avi V, Afilalo J, Armstrong A, Ernande L et al Recommendations for cardiac chamber quantification by echocardiography in adults: an update from the American Society of echocardiography and the European Association of cardiovascular Imaging. Eur Heart J Cardiovasc Imaging 2015;16:233–70. 25712077 10.1093/ehjci/jev014

[25] Sherrid MV, Wever-Pinzon O, Shah A, Chaudhry FA. Reflections of inflections in hypertrophic cardiomyopathy. J Am Coll Cardiol 2009;54:212–9. 19589433 10.1016/j.jacc.2009.03.052

[26] Bland JM, Altman DG. Calculating correlation coefficients with repeated observations: part 2–correlation between subjects. BMJ 1995;310:633. 7703752 10.1136/bmj.310.6980.633 PMC2549010

[27] Bland JM, Altman DG. Correlation, regression, and repeated data. BMJ 1994;308:896. 8173371 10.1136/bmj.308.6933.896 PMC2539813

[28] Ciampi Q, Olivotto I, Gardini C, Mori F, Peteiro J, Monserrat L et al Prognostic role of stress echocardiography in hypertrophic cardiomyopathy: the international stress Echo registry. Int J Cardiol 2016;219:331–8. 27348413 10.1016/j.ijcard.2016.06.044

[29] Clarke NR, Timperley J, Kelion AD, Banning AP. Transthoracic echocardiography using second harmonic imaging with Valsalva manoeuvre for the detection of right to left shunts. Eur J Echocardiogr 2004;5:176–81. 15147659 10.1016/S1525-2167(03)00076-3

[30] Rodrigues AC, Picard MH, Carbone A, Arruda AL, Flores T, Klohn J et al Importance of adequately performed Valsalva maneuver to detect patent foramen ovale during transesophageal echocardiography. J Am Soc Echocardiogr 2013;26:1337–43. 23993693 10.1016/j.echo.2013.07.016

[31] Hudson LD, Slutsky AS. Acute Respiratory Failure Goldman’s Cecil Medicine. Amsterdam: Elsevier; 2012. p629–38.

[32] Takaya Y, Watanabe N, Ikeda M, Akagi T, Nakayama R, Nakagawa K et al Importance of abdominal compression Valsalva maneuver and microbubble grading in contrast transthoracic echocardiography. J Am Soc Echocardiogr 2020;33:201–6. 31837927 10.1016/j.echo.2019.09.018

