# Supplementary material for: Feasibility of party balloon inflation manoeuvre for haemodynamic provocation: a pilot study in healthy volunteers
Source: Eur Heart J Imaging Methods Pract. 2025 Jun 3;3(1):qyaf071. doi: 10.1093/ehjimp/qyaf071 (PMC12168156; doi:10.1093/ehjimp/qyaf071)
Supplement: qyaf071_Supplementary_Data [file qyaf071_supplementary_data.docx]

**SUPPLEMENTARY MATERIAL**

**Supplementary Figures**

**Supplementary Figure 1. Syringe barrel connected to an anaeroid manometer**


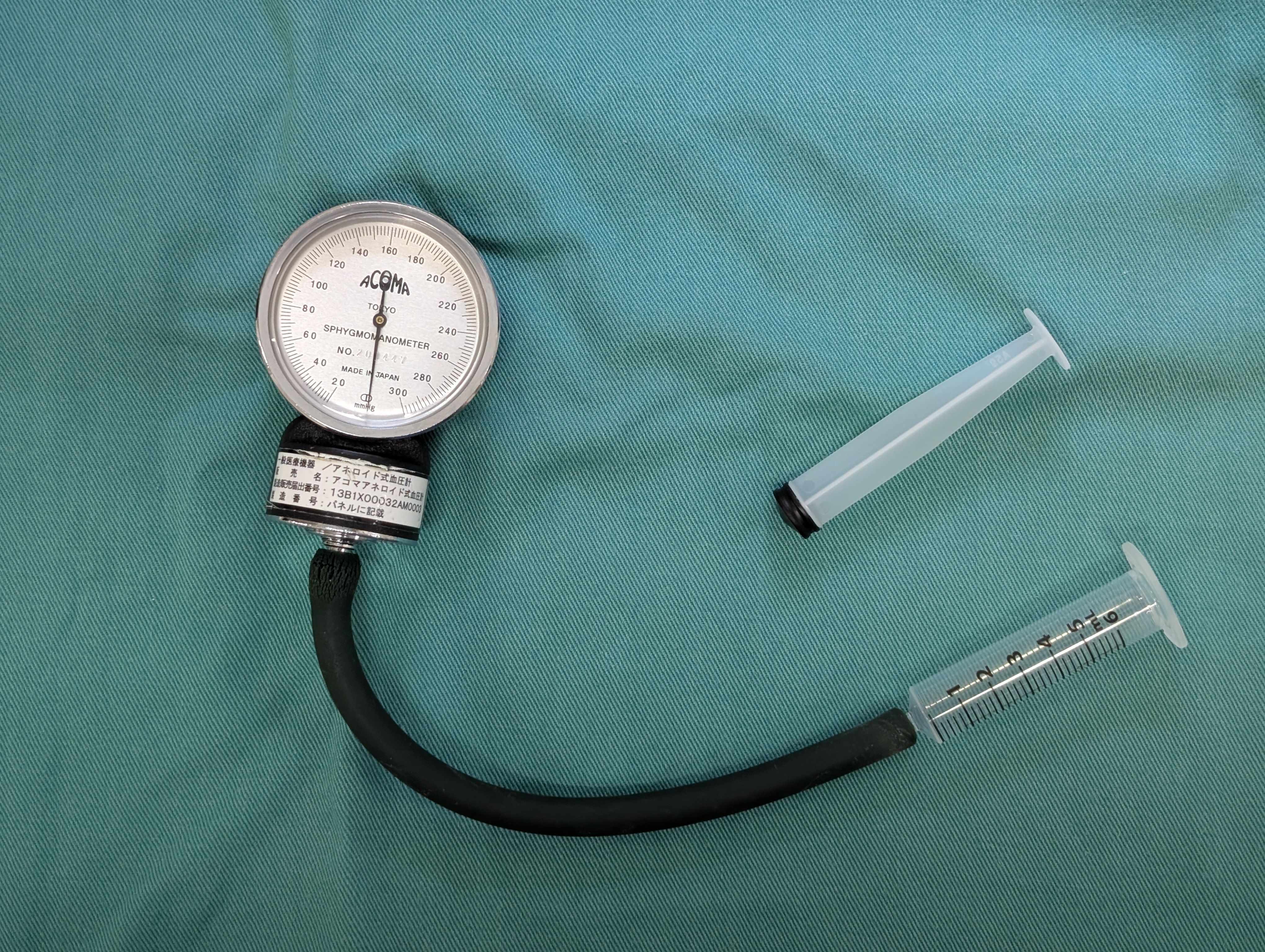


**Supplementary Figure 2. Comparison of LVEDV (A) and Borg RPE scale (B) during rest, PBIM, and GDVM**


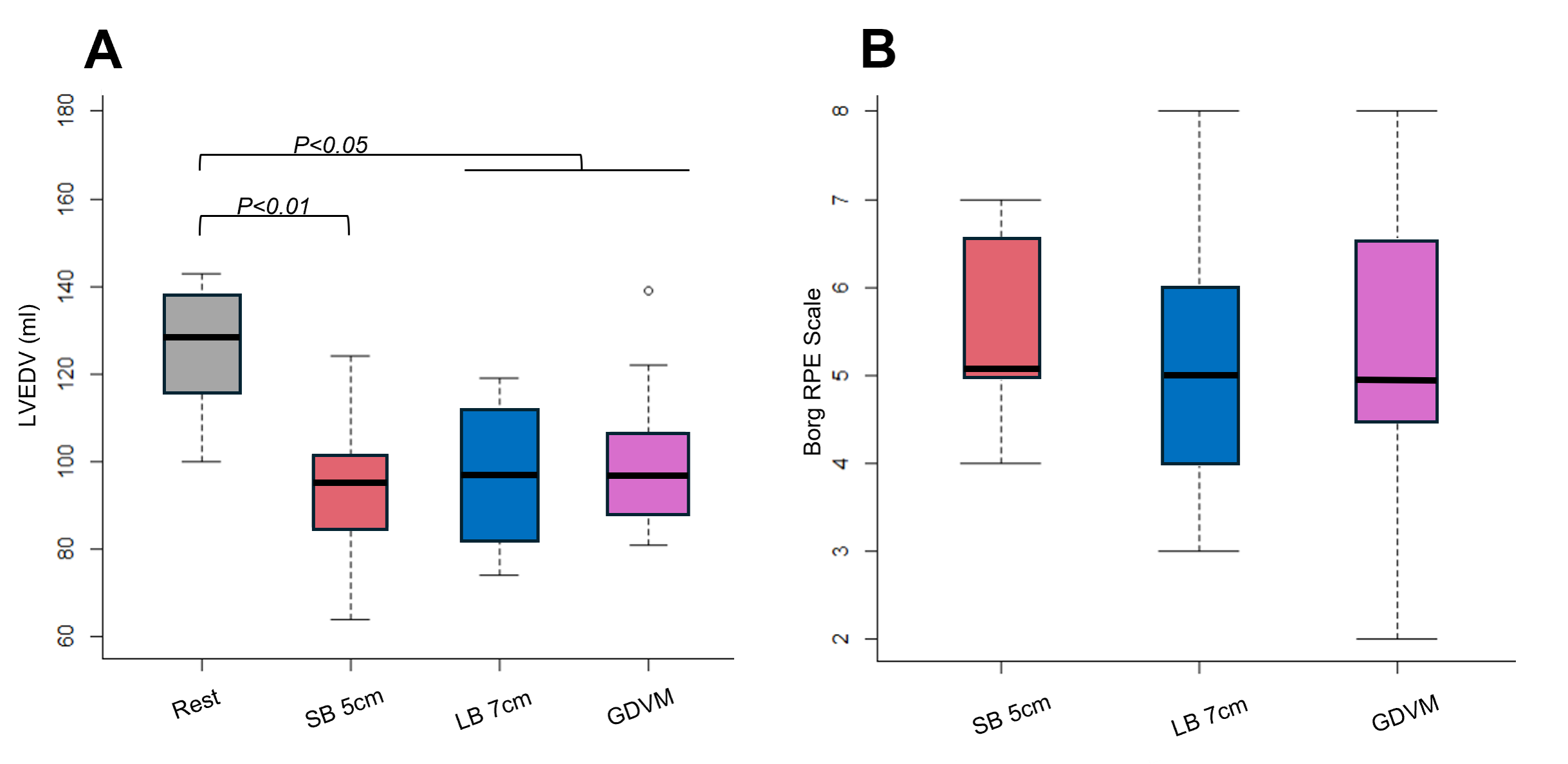


Within each box plot, the horizontal black lines denote the median values. The boxes extend from the 25th to the 75th percentile of each group's distribution of values. The vertical lines represent adjacent values (i.e., the most extreme values within 1.5 times the interquartile range of the 25th and 75th percentiles of each group). Dots denote observations outside the range of adjacent values (outliers).

**Abbreviations**: GDVM, goal-directed Valsalva manoeuvre; LB, larger balloon; LVEDV, left ventricular end-diastolic volume; RPE, Rating of Perceived Exertion; SB, smaller balloon

**Supplementary Table 1:** Participant for characteristics and baseline echocardiographic parameters

| Age, years | 32.0 (29–34) |
| --- | --- |
| Male, % | 12 (100) |
| Weight, kg | 63.5 (61–67) |
| Height, cm | 173.0 (171–176) |
| BSA, cm^2^ | 1.70 (1.70–1.83) |
| Rest HR, bpm | 70.0 (58.0–74.0) |
| LVDd, mm | 48.9 (41.6–47.1) |
| LVDs, mm | 29.8 (27.9–31.4) |
| LVEDV index, ml/m^2^ | 46.5 (41.2–54.2) |
| LVESV index, ml/m^2^ | 17.4 (14.8–18.2) |
| LVEF, % | 61.5 (55.1–64.5) |
| LAV index, ml/m^2^ | 14.6 (11.2–21.5) |
| E/e' | 4.3 (4.3–5.4) |
| MR: none / trace / >mild | 10 (83) / 2 (17) / 0 (0) |
| TR: none / trace / >mild | 12 (100) / 0 (0) / 0 (0) |

Values are median (interquartile range) or n (%).

BP, blood pressure; BSA, body surface area; HR, heart rate; LVDd, left ventricular end-diastolic dimension; LVDs, left ventricular diastolic diameter; LVEF, left ventricular ejection fraction, LAV, left atrial volume; E, transmitral peak E-Wave velocity; e', peak diastolic annular velocity; MR, mitral regurgitation; TR, tricuspid regurgitation.
